# Questioning the value of present life: The lived experience of older people who see no future for themselves

**DOI:** 10.1080/13607863.2023.2197850

**Published:** 2023-04-10

**Authors:** Vera E. van den Berg, Thessa W. Thölking, Carlo J. W. Leget, Iris D. Hartog, Margot L. Zomers, Johannes J. M. van Delden, Els J. van Wijngaarden

**Affiliations:** a Care Ethics, University of Humanistic Studies, Utrecht, the Netherlands; b Department of Anesthesiology, Pain and Palliative Medicine, Contemporary Meanings of Ageing and Dying, Radboud University Medical Center Nijmegen, Nijmegen, the Netherlands; c Department of Care Ethics, University of Humanistic Studies, Utrecht, the Netherlands; d Center of Expertise of Palliative Care, Leiden University Medical Center, Leiden, The Netherlands; e Comprehensive Cancer Organisation Utrecht, Utrecht, the Netherlands; f Julius Center for Health Sciences and Primary Care, University Medical Center Utrecht, Utrecht, the Netherlands

**Keywords:** older people, aging, seeing no future, everyday loneliness, interviews, phenomenological method

## Abstract

**Objectives:**

To describe the lived experience of older people who see no future for oneself in the context of aging and the possible development of a wish to die.

**Methods:**

Data were collected from 34 interviews with people of 55-92 years. A phenomenological hermeneutical analysis was performed using crafted stories as an analytical device.

**Results:**

Four intertwined constituents together with the essence of the phenomenon provide a layered description of what it means to see no future for oneself. In all constituents: 1) not sharing everyday life, 2) looking for new commitments, 3) facing present losses and future fears and 4) imagining not waking up in the morning, the essence losing zest for life seeped through their daily experiences.

**Conclusions:**

As their horizon of future possibilities is shrinking, older people in our study experience a loss of zest for life and start to questioning the value of their present lives. And although a certain languishing mood can be discovered, the phenomenon ‘seeing no future for oneself’ does not entail a wish to die.

## Introduction

An aging population has become a demographic trend in many societies around the world and due to great advances in healthcare, more people reach deep old age. In Western societies there is a persuasive focus on positive aging where people are served an ideal to age as healthy, active and independent as possible (Rowe and Kahn 1997, 2015). By promoting such a perspective on aging, there is less attention for the vulnerabilities and difficulties that come with aging and age itself (Gilleard 2018). In parallel, society also holds stereotype images of old age as an undesirable state of decline filled with loneliness and meaninglessness (Laceulle and Baars 2014). For some people these negative stereotypes may lead to an internalized view of burdensomeness and a negative outlook on the future contributing to a lack of meaning in life (Van Orden et al. 2012). In fact, there is a group who feel that life is no longer meaningful and a small part of them even develop a wish to die (WTD) without being severely ill (Hartog et al. 2020; Van Wijngaarden, Leget, and Goossensen 2015).

A wish to die can be described as longing for death to come for oneself (Elzinga et al. 2022). Expressing a wish to die is not necessarily unequivocal and can have different intentions, meanings and functions (Ohnsorge, Gudat, and Rehmann-Sutter 2014; Ohnsorge, Gudat, and Rehmann-Sutter 2014). Furthermore, it comes with various ambivalent feelings fluctuating in stability and intensity (Van Wijngaarden et al. 2021). For example, the WTD can be experienced at the same time with a wish to live, it can change over time and it can disappear. It has been suggested that a WTD of persons who are not severely ill, is mainly associated with ‘completed life’ ‘tiredness of life’, meaninglessness and worthlessness (Van Den Noortgate and Van Humbeeck 2021).

The study of wishes to die is mainly centred in the field of mental health, suicide and suicidal ideation or in relation to terminal illness. Because research into wishes to die is predominantly conducted in these fields there is an observable gap in understanding how a WTD may be experienced in the context of aging. In other words, we need to extend our understanding of (the experience of) a WTD beyond the medical perspective. Also in later life, a WTD is mostly interpreted and explained as a symptom of clinical depression and suicidal ideation (Harwood et al. 2006; Jorm et al. 1995; Raue et al. 2007) and is considered to be a growing universal health problem (Bonnewyn 2016). The question has risen whether a WTD should only be considered as a symptom of underlying psychopathology (Szanto et al. 2013) or whether it might also be considered as a phenomenon that comes with aging (McCue et al. 2015; Rossom et al. 2019; Van Wijngaarden, Leget, and Goossensen 2015).

In the Netherlands there is a particular interest for the WTD outside the medical domain in the context of the ongoing debate whether older people who are not severely ill should receive aid in dying (Hartog et al. 2020; Kox et al. 2021). In this so called ‘completed life’ debate the older people concerned are referred to as people, mostly of old age, who at their own judgement see no future for themselves and as a result develop a persistent active wish to die without suffering that (mainly) originates in a medically classifiable condition (Schnabel et al. 2016). It remains unclear, however, how ‘seeing no future for oneself’ should be interpreted from a insiders perspective and how it may relate to a wish to die.

Future perspective has been extensively studied in the field of psychology as an element of ‘time perspective’, which refers to how individuals perceive, evaluate and relate to the past and the future (Desmyter and De Raedt 2012; Fung and Isaacowitz 2016; Lang and Carstensen 2002). Research examining the relationship between wellbeing and time perspective in older people indicates an age-related shift from concerns about the future to concerns about the present (Åström et al. 2019; Brothers et al. 2016). These studies show that thinking about what the future beholds can be a source of distress for older people (Åström et al. 2019; Lang and Carstensen 2002).What they seldom portray is a deepening of how it is to live with this changing future perspective. Therefore, in this study we explore this concept of ‘seeing no future for oneself’, as a lived experience in the context of aging.

With this study we aim to understand what it means for older people to live while seeing no future for themselves. To access the lived experience, we conducted a phenomenological interview study, as part of a larger study that examined wishes to die of older persons who see no future for themselves and who may develop a persistent wish to die without being severely ill. The central guiding question of the interview study was: What does it mean for older people to see no future for oneself?

## Method

### Phenomenological research design

In order to describe the lived experiences of the participants, we chose a phenomenological approach, exploring human experience as it is lived through rather than how it is conceptualized or theorized (Van Manen 2016).The focus is not on causal explanations or reasons but on the lived experience of the phenomenon. What empirical phenomenologists aspire to describe are the implicit meaning structures of a lived experience. Things do not appear to us as simply just there, but appear in particular ways for example as repellent or out of place (Carel 2016). In order to understand the phenomenon of seeing no future for oneself more closely, we concentrate on how this phenomenon presents itself to those who live it (Van der Meide et al. 2020). The phenomenological question of this study was: What is the meaning structure of the lived experience of older people who see no future for oneself?

#### Context and recruitment

This research was part of the PERSPECTIVE-study which was conducted in 2019 by an interdisciplinary team commissioned by the Dutch Ministry of Health, Welfare and Sport. In the PERSPECTIVE-study an extensive survey among 32.477 Dutch citizens aged 55 and older was used to gain insight in the prevalence of the group of people who have a persistent wish to die without being severely ill (Hartog et al. 2020).

At the end of this survey, respondents were asked whether at that moment they recognized themselves in the qualifications: ‘seeing no future for oneself, longing for death while not being severely ill’. When they gave an affirmative answer, respondents could leave their contact details for an interview, if they were willing to participate.

#### Pre-selection/selection

A total of 171 respondents who recognized themselves in the selection-question, gave their consent to be called for an interview. All potential participants were called but because 70 persons could not be reached a pre-selection interview by telephone was held with 101 persons see flowchart (appendix). Using a guideline to remind them of the topic of the study and to check whether this still applied to them semi-structured questions were asked. For example, whether the potential respondents saw any future for themselves, how they viewed their perspective on life, and if they sometimes longed for death to come. In addition, other characteristics were gathered such as gender, age, living arrangement, social network and health. These preselection interviews lasted between 3 and 45 min.

To get a rich and in-depth description of the phenomenon (or experiences) we searched for variety among the participants. Therefore, we conducted purposeful sampling among the 101 preselected participants and chose 50 participants. The selection was made based on two criteria that were relevant to answer the question under study (Patton 2014): 1) the experience of seeing no future for oneself, and if applicable 2) the possible experience of a wish to die. To find a rich variety we also made a selection on demographic characteristics giving priority to participants older than 80 years. The final selection was discussed by all eight members of the research team.

Respondents were excluded when they wanted to participate mainly in order to express their opinion or were at that moment under treatment for a life-threatening disease or mental disorder. Of the 50 selected participants 16 withdrew themselves during different stages of the study because of practical reasons or the study subject.

#### Interviews

Between May and August 2019, 34 interviews were conducted by VBX1, IH and MZ, using an interview guide. The interviews lasted between 26 and 240 min with an average of 96 min.

The focus of the interviews was on participants’ experiences, and in order to offer a starting point for dialogue the following case description which was also used in the survey to select participants for this study, was read out loud by the interviewer.

Mrs P is 76 years old and feels very lonely. Her husband died a year ago. She has lovely children and a couple of close friends, but they cannot fill in the emptiness of missing her husband. She is physically and mentally healthy but doesn’t see a future for herself. The beautiful years lie behind her. She finds her life completed and would rather be dead.

After the reading, participants were asked to what extent they could relate to this case-description. Did they recognize themselves and in what parts specifically? In order to gain (access to) experiences, the interviewers invited the participant to describe moments or situations that evoked these experiences. For example: can you describe a moment when you experienced seeing no future for yourself or a moment when you experienced a diminishing perspective? The conversation that unfolded led to follow-up questions such as: could you tell us more about this? When was that, what happened, how did you feel? The interviews were audio recorded and transcribed. After each interview the interviewers wrote reflective notes in a reflective diary about their impressions of the setting, participant and impressions of the interview as a whole.

After the interview, the participant answered a short survey with a few demographic questions. To explore the general health and well-being of the participants the EQ5D. a health-related quality of life questionnaire was completed (Herdman et al. 2011). For the screening of a potential diagnosis of depression we used the Hospital Anxiety and Depression Scale (HADS-D) (Zigmond and Snaith 1983).

##### Data analysis

The aim of phenomenological analysis is to expose meanings from a phenomenon by closely studying individual experiences to reveal common features of experiences by adopting an open passive attitude of wonder (Van der Meide 2018; Van Manen 2016).

In this study we adhered to the following five steps (Crowther et al. 2017; Van der Meide 2018):

1. Slowing down pre-emptive understanding by (re-)reading the transcripts and the reflexive notes of the interviews while listening to the audio files. During this step, reflections, questions and thoughts were documented and discussed in the team. In order to stimulate intersubjectivity the interviewers read and listened to one interview conducted by a colleague interviewer next to their own interviews.
2. Composing textual portraits from the individual transcripts in which parts of the interviews that contained descriptions of experiences of ‘seeing no future for oneself’ were compiled without changing the actual content and meaning of the text (Crowther et al. 2017).
3. Coming to a phenomenological thematization of all textual portraits, each interview was analyzed for themes. Because of the large number of portraits themes were identified and grouped using ATLAS ti. Version 8.XXX. In order to enhance intersubjectivity at the start of this step, one interview was analyzed together by researchers VB, IH, MZ and EW. The theme analyses of the remaining interviews were divided by VB, IH, MZ and TT.
4. Describing the themes to explicate the phenomenon ‘seeing no future for oneself’. VB and TT merged the themes by reflective writing with aid of the textual portraits aiming to reveal the phenomenon.
5. Finalizing the different themes and developments by reflective phenomenological writing and reading (Van Manen 2016).

## Ethical consideration and positioning

This study was part of a large research proposal that was evaluated by the Review Ethics Committee of the UMC Utrecht (METC). They concluded that the study did not fall under the scope of the Dutch Act on Medical Research Involving Human Subject (WMO) and was therefore exempt from review (METC protocol number: 19-156/C). All ethical aspects of responsible research such as confidentially, protection of privacy, accurate information and written consent were addressed with the utmost care. Participants had the right to withdraw without reason at any time.

In order to ensure the safety and well-being of the persons in our study, the subjects of wishes to die, suicidal ideation and suicide were discussed with a Dutch research and suicide prevention organization ‘113’ during the study design phase. Experts from this organization indicated that interviewing persons about death wishes and suicide was not harmful. With insights from this consultation a precautionary safety protocol was drawn up for cases in which researchers had the impression that a participant was in acute distress or need. This was never the case, however.

For a phenomenological research it is also important to have an engaged reflective stance and therefore explicate our position in relation to this study (Crowther and Thomson 2020). In this study all researchers were from the Netherlands and had a background in ethics. Except for one researcher with bicultural roots, all were white. Besides two men over 55 years, the rest of the team consisted of women under 45 years.

## Results

**Figure 1.**
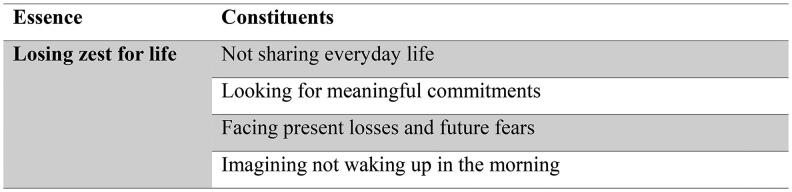
Essence of the experience of seeing no future for oneself and its constituents.

In this section, we will sequently describe the essence of the experience seeing no future for oneself and the constituents that further illuminate the different aspects of this experience as shown in Figure 1 (Dahlberg 2006). Although the constituents are described separately, they are intertwined and in conjunction with the essence provide a layered description of what it means to live while seeing no future for oneself.

Demographic characteristics and general health and well-being parameters of the study population are presented in Table 1.

**Table 1.** Characteristics of the participants.

Characteristics	
**Gender**	*N* ** = 34**
Male	13
female	21
**Age**	*N* ** = 34**
55–60	4
61–65	5
66–70	4
71–75	11
76–80	5
81–85	3
86–90	2
Average age	71
**Marital status**	*N* ** = 34**
Partner (married and/or living together)	6
Partner (partner living in residential care home)	1
Partner (not living together)	1
Widowed	11
Divorced	9
No partner	6
**Children**	*N* ** = 34**
No children	7
1 child	3
2 children	19
3 children or more	5
**Living condition**	*N* ** = 34**
Independent	33
Independent in a service apartment	1
**Belief**	*N* ** = 34**
Humanism	1
Christianity	10
Spiritual	5
Agnosticism	1
No belief	17
**Education**	*N* ** = 34**
High school	9
Intermediate vocational education	10
Higher vocational education	12
University	1
Not mentioned	2
**Relevant health problems mentioned by participants**	*N* ** = 34** ^1^
None	11
Pain	7
Cardiac failure	5
Neuropathy/rheumatoid arthritis	5
Psychological problems	2
COPD	1
Intestinal disease	2
Physical deterioration	3
Physical disability by loss of eye/leg	2
**HADS-D** * **score**	*N* ** = 34**
1–7 (no indication)	14
8–10 (mild)	8
11–15 (moderate)	11
16 or above (severe)	1
**VAS** ** **score (1-10)**	*N* ** = 34**
1	–
2	–
3	1
4	8
5	2
6	4
7	7
8	8
9	2
10	2
**EQ5D score** *** **(5-25)**	
5–10	20
11–15	9
16–20	5
20+	–

*HADS-D: the depression subscale of the Hospital and Anxiety Depression Scale (Dutch version).

** VAS: the Visual Analogue Scale.

*** EQ5D: a self-assessed, health related quality of life questionnaire (Dutch version).

### Losing zest for life

The meaning of seeing no future for oneself emerged as an existential phenomenon and can be understood as losing zest for life. It is characterized by moments of silence and standstill in which the value and meaning of present life was questioned. This evoked an uneasy feeling among the participants in which the loss of zest for life was mostly felt.

These questions, thoughts and experiences of ‘seeing no future for oneself’ were expressed with vague and indefinite terms often using the word ‘it’ for example: ‘*as long as I am busy*, *I don’t feel it’* or ‘*during summer it is fine’.* Although, it remained unclear to what the word ‘it’ exactly referred to or what it precisely meant, it revealed an overall mood or feeling.

Other frequently used expressions were: ‘*There is no need’, ‘Don’t bother’,* and ‘*If I won’t wake up tomorrow, that would be fine’.* By using these ambiguous expressions and words, participants were able to talk about their experience and at the same time it showed the difficulty and elusiveness of it.

Besides the questioning, a mode of staying active and keeping oneself busy in the present played a fundamental role in evading moments of emptiness that evoked this questioning of the value of present life. Caught in these moments of emptiness, they reflected on what they felt, thought and experienced. For example: ‘*Why am I doing this and for what reason?’ ‘Is this all?’ ‘Now what?’* and ‘*Why do I wake up every day*? The questioning about present life, withheld participants to actively engage with or think about future plans.

Participants seldom spoke firmly when they talked about their experience of seeing no future for oneself but rather in a doubting, seeking manner. In addition to the many questions, a tone of resignation could be observed. Participants talked about their experiences as something they just lived with, and not something they should or wanted to change or take action on.

This doubting indecisive tone of voice was contrasted by the active and slightly more decisive tone of the few participants with a more defined idea of the future due to a progressive disease. ‘*All those pains, when does it stop? If I cannot make myself a cup of coffee anymore then it’s over’ (p.17).*

The few participants who also spoke with a slightly different tone of voice, were those with a new partner. Finding a new partner opened up new possibilities and they spoke in a careful though lively manner about the future for example: planning holidays and moving to new houses.

### Not sharing everyday life

The majority of the participants had no partner; some of them were widowed, others were divorced and a small number had always been single. Despite the fact that most participants had a social network which often included their children they could rely on, the idea of having to face a future alone and by themselves, made them say: ‘*I have no future either’.*

Loneliness played a big role in their lives. Being alone in an ‘empty house’ colored the experiences of everyday life in which the silence also felt like a void. The radio or television were often switched on during the days and in one interview the participant felt uncomfortable to switch the radio off during the interview. ‘*That is a part of life here as well. I have all radios synchronized in the house. As soon as I come home, the moment I wake up, I turn on the radio’. (p.20)*

Various times during the day were mentioned as particularly difficult and the experience of everyday loneliness was mostly felt during these moments. Some participants referred to the mornings, stating ‘*there is no reason to stay in bed’* when not waking up next to a loved one. Others mentioned the evenings, coming home to a house ‘*where nobody is waiting for you’* or eating alone not only evoked feelings of meaninglessness and loneliness but also started questioning life in the present.

In the mornings, when I am busy, I don’t feel **it**. But in the afternoon when I am really tired because of my weary back, I can hardly walk the dog, I lie down for a minute and it starts. Then I have this really strange feeling: ‘what am I actually doing here?’ (p.34)

Weekends, ‘*Sundays are the worst’* and holidays were also recurring topics which were considered to be difficult to give structure or meaning to. Participants seemed keen to actively make plans for these particular moments in the nearby future.

I am always alone. All the holidays I am alone. On Christmas, on Easter, on Whitsun weekend. Already for 13 years, 13 times New Year’s Eve, all alone. That’s no fun. This year for the first time alone on my birthday. It was on a Monday which is a bit of a bad day for working people, but still. No call, nor emails. I got an email from the insurance company: happy birthday. But from family, I heard absolutely nothing. (p.11)

Though participants had children and they perceived the relationship as good, the experience of not being able to share everyday life was tough. Participants were aware of the weight the subject of loneliness could have on their social relationships. In conversations they sometimes avoided the subject or only mentioned it briefly in order to keep the relation good and in balance.

The importance of sharing a life with somebody was illustrated by the few widowed participants who found a new relationship. They experienced less everyday loneliness, especially in how daily activities such as cooking, having dinner and weekend getaways were perceived.

I have a girlfriend; we’re taking it slow. I don’t want to be alone. We get along quite well, the only thing is, she has an opinion on pretty much everything. But when she is not around, I miss her. I would rather have her bullshit than be here on my own. Look, I like to cook, but not for myself. When she is coming over, I make something special. (p.20)

At the same time, it did not necessarily mean that these participants saw more future possibilities nor that they made plans for the near or far future because the new relationship came with new doubts and ambivalences.

Well, look, every life phase comes with change, it brings nice things but also other problems. In that sense, how will it (the relationship) develop? I mean there is someone new, a man who is a bit older than me. He lives 85 miles away, so as long as we both still can drive, that is no problem. But what about the future, do I want to live together? What will be the consequences? That sort of questions, again. Of course, new challenges but also new problems. (p.7)

### Looking for meaningful commitments

Besides the loneliness in everyday life, the absence of work obligations or care responsibilities gave rise to the questioning of the value of life ‘*Is this it’* or ‘*Is this all?’* Although, participants gave themselves all kind of chores or duties to stay busy during the days, the reflection of ‘is this it?’ came along with a feeling of loss of purpose and a sense of not mattering. Not only participants who were on retirement, but also the few who were still working, had this idea of ‘*as long as I keep myself busy then it’s okay’.* The prospect of retirement without work obligations was not something they looked forward to: ‘*work is my form of occupational therapy. I worry about what’s to come’ (p.6)*.

Participants filled their daily lives by taking up hobbies or doing volunteer work. But volunteer work also gave rise to new questions ‘*do I really contribute with this*’ or ‘*how much responsibility can volunteer work ask from me?*’ Although volunteer work could add to a sense of purpose for some participants, the idea that activities were in a way noncommittal did not contribute to the feeling of acknowledgement and being valued. This noncommittal nature of volunteer work underlined the lack of being needed and enhanced the experience of uselessness and meaninglessness evoking the question ‘*why bother?*’

It is like as if everything has two sides. It used to be different, when it only was one-sided. I had to go to work. Work didn’t know days of weariness. Now I don’t have to. I can but I don’t have to. The freedom is in a way really nice. But the fact that I don’t necessarily have to, also means that it’s just less important. I don’t feel that important anymore, not so appreciated anymore, not so useful as before. And that feels like a black hole. (p.31)

In contrast to volunteer work, that came with ambivalences, the experience of caring for someone or something did contribute to the feeling of being needed. Participants gave different examples of persons and things they took care of or chose to be committed to. A spouse or a sick friend, a pet or a garden all were asking for commitment. This self-imposed commitment of caring was something to live for and a reason to postpone questioning on the value of life or even thoughts of a latent wish to die: ‘*if my wife gets better, then I want to grow old a little bit more’. (p.19)* They experienced a certain renewed zest for life because of this personal dedication, for example to play a role in the upbringing of a grandchild with whom a special connection was felt. In this act of caring for someone or something, they experienced a sense of mattering.

First I thought: I quit (living), why bother? But then I thought of my granddaughter. She still needs me. She is just 24. Until Sunday she had never expressed that to me. So now I want to stay, I still have this duty to do. (p.9)

Other participants were more doubtful whether to take up the responsibility of caring for something especially when they were considering adopting a new pet: ‘*how many years do I have?’* Although they had experienced that a pet in their lives, besides company also gave them a reason to live, their limited life expectancy withheld them to make this commitment in the present.

### Facing present losses and future fears

Not only the limited amount of years ahead gave rise to the experience of seeing no future for oneself, but also different forms of loss in the present time evoked questioning the value of life. Losing a spouse, a close friend, but also losing certain skills changed everyday life and affected participants identity. A few mentioned that by losing their partner, they lost their social life and they had difficulties to deal with their new marital status. With this loss, they not only lost a future but also a shared past, a life built together.

Going on retirement was another life event that meant losing status, a network and connection with the world. It made the person’s world smaller which sometimes led to feelings of uselessness and the question: ‘*Is this all?’*

Also, the experience of losing certain skills in the present could contribute to a developing fear of the future. In a few interviews this was related to own experiences of declining physical health which for example resulted in loss of mobility ‘*And now I cannot drive anymore, it is horrible my car is my everything, it is my freedom*’ *(p.11).* Fears for the future were often fears of losing independence.

Besides contributing to fear of the future, a declining health could also serve as a starting point for reflecting and pondering about the future. The consideration to have a hip surgery or cardiac surgery, probably improving wellbeing and health, raised new worries. Being dependent while recovering gave enough reasons to doubt whether to have the operation in the first place. Despite acknowledging the positive aspects of these possibilities, they kept questioning and postponing the decision.

Look, I have this cardiac problem, it is not that bad yet. I can still take the bike, and take the stairs if I have to. But I am really discussing it with myself: is this surgery really necessary? Do I really want it? For whom? If you will go for surgery, then it should benefit my life. But if that life is already often meaningless, why should I even consider surgery? (p.14)

In other interviews this fear was connected with the fear of elderly care in general and fear of ending up in a nursing home. This fear was sometimes based on seeing others (often a close one) in this kind of situations.

My sister is older than me, and she has dementia. I pity her a lot. She is in a nursing home, nearby. Horrible. That is something I absolutely do not want to experience. I do not want to die that way. That is the reason why I say: boy, when that time comes, I will end it. (p.26).

Many participants connected a potential undesirable future with the development of an anticipated wish to die. This scenario was addressed in an ‘if…then- construction; if a certain occasion would occur, then they would rather die. Anticipating this future scenario, some participants prepared arrangements to prevent prolonging their lives. They wore a do-not-resuscitate token or wrote an advanced directive.

In addition, many participants were positive about the idea of introducing and legalizing an ‘suicide pill’ for older people. With the ‘suicide pill’ they referred to certain pharmaceutical means which could lead to a gentle death at a self-chosen moment. This ‘suicide pill’ does not currently exist as a legal option but some pharmaceuticals can be obtained illegally.

The thought of emptying my house in order to move…then I sometimes wish; I rather have a hand full of (morphine) pills. (p.11)

The idea of having this ‘suicide pill’ that they could take when the if-then-scenario would occur, would reassure most of them. At the same time, some participants foresaw new dilemmas looming.

That pill (suicide pill) is not for now, but I can imagine that I would want it in the future. I realize how complex that is. Look, sometimes I have three days that I feel depressed. And suddenly something happens that makes life more beautiful and the depression disappears. Supposedly, I would have such a pill, maybe I would have used in in one of those three days. But a couple of days later I’m in heaven, then I might regret it. Which moment do you decide? (p.14)

### Imagining not waking up in the morning

In contrast and parallel to the idea of actively taking a ‘suicide pill’ when the time comes, many participants also mentioned the scenario of not waking up in the morning or in some cases not wanting to wake up in the morning. Imagining not waking up in the morning pointed to an ideal way of dying. This scenario represented to two elements: it referred to both a (sudden) unplanned and natural way of dying. A natural death equaled to a peaceful death, a good way of dying without suffering.

The tone of voice in which participants spoke about not wanting to wake up, differed. Some participants experienced a strong desire not to wake up anymore, often related to physical suffering which was caused by a medical condition. ‘*When the pain gets worse, I ask myself, why do I wake up every morning?!*’ *(p.17)* Others were more indifferent. ‘*Since a long time, it would be fine if I wouldn’t wake up in the mornings.’ (p.6)*

Some participants described specific moments in which they had preliminary thoughts about death and dying. The weather, the season, or particular times of the day, for example the evening or the morning, stirred up this thought of not wanting to wake up.

In the winter, I am always alone, sitting here by myself all the time. The weather is always depressing and gloomy. Not a spark of sunshine, itis making me depressed. And then I think “what am I doing here? and, “just shoot me to death”. The winter is horrible. Give me half a suicide pill, so I can wake up in April. Not a whole pill, because then I wouldn’t be there anymore. (p.2)

Although at two moments during the selection process participants had affirmed having a WTD, at the time they were interviewed, all participants did not have a current wish to die. A few participants had longed for death in an earlier time of their lives, others spoke about moments in the future in which they would rather die.

However, sometimes a diminishing wish to live and glimpses of a latent WTD in specific situations could be perceived through the indecisive and indifferent tone most participants spoke with and their thoughts of not waking up in the morning.

## Discussion

The purpose of this study was to explore the lived experience of seeing no future for oneself as it is lived and experienced by older people who are not severely ill. The findings of our study reveal four intertwined constituents: not sharing everyday life, looking for meaningful commitments, facing presents losses and future fears and imagining not waking up in the morning. These constituents contribute to the essential meaning that is losing zest for life against a background of diminishing future possibilities in later life.

Our findings reflect the phenomenological idea that the future is not an objective period of time-to-come but a dimension that shapes what is meaningful in the present. The present envelops the future. That is, present occurrences and circumstances get their meaning out of future purpose (Giorgi 2015; Van den Berg 1972). If there is no sense of a future purpose, the present can be experienced as empty and less meaningful as reflected in the stories of our participants.

That present and future are closely connected and mutually influence one and other, is also highlighted by our findings: when the present is experienced as empty and meaningless, present life is questioned. This close relation between future and present was reflected in the themes not sharing everyday life and facing present losses and future fears. It supports the idea that in order to live in the present as a mode of well-being, worries about the future should be bracketed (Carel 2016).

The importance of a sense of purpose is also reflected in the theme looking for meaningful commitments. Participants experienced a sense of purpose and a sense being needed when taking care for someone or something. By making this commitment they connected with and opened up for future possibilities. This is in line with the phenomenological understanding that as human beings we are always constituted by our movement into future possibilities (Johnson 2000) .

Being in the world as temporality reminds us that all meaning and discovery are fraught with finitude (Carel 2016). Aging has a close relationship to both dying and death. Moreover, our findings elucidate that there are different ways of being in relation with the understanding life as finite. As humans we are always living into future possibilities, we are having concerns about and investments in ourselves, other things and other people.

The difficulty to give voice to complex elusive experiences is reflected in the use of the ambiguous word ‘it’. Although, it remains unclear to what the word ‘it’ precisely refers to, it reveals an overall mood of loss of zest for life. The reflections which come up with this mood concern daily life in the present and are less about reminiscing life as a whole.

The current study also gives a broader insight in several psychological concepts, in which light the findings can be discussed. Coming from the field of positive psychology the concept zest for life can be defined as being satisfied with different aspects of one’s life, including the will to live and enjoyment of life. It also refers to having energy and courage to look for new experiences and is perceived as an important aspect of living and aging well (Collins et al. 2016; Glasberg, Pellfolk, and Fagerström 2014).

Although our participants of whom the majority were in their seventies, still had energy and were content with several aspects of their lives, a diminishing vitality and enjoyment seeped through their stories. They reflected about their present lives and started questioning the value of it but not as an active anticipation of an unwanted future but more as a result of an alienated feeling which they felt in moments of emptiness. With their focus on keeping on going, they navigated through everyday life by staying busy with activities. Most of them neither made plans for the future nor had aspirations for the years to come. In fact, future possibilities were strongly connected with future difficulties as reflected in their future fears. By staying busy in the present, they kept the future at bay.

Of interest are some characteristics of the participants that raise questions concerning the association with depression. It is noteworthy that for almost half of the participants there was no indication for possible depression whereas for a small majority there were indications for a mild or moderate depression. Only for one participant there were indications of a more severe depression (see Table 1 Characteristics of the participants).

In combination with their indecisive, doubting but also resigned tone of voice, it can be argued that the participants of this study are experiencing an absence of mental health also defined as the concept of languishing. People with a languishing mood experience emptiness and stagnation, constituting a life of quiet despair (Keyes 2002).

Although, only a few participants of our study were living a life of quiet despair, all participants struggled with several aspects of life. The different constituents found in this study were closely connected with difficulties that come with old age and aging itself such as a decline in physical heath, changing social relations, narrowing of life’s opportunities and the proximity of death (Gilleard 2018) . Moreover, loneliness, age related loss and fears are known to contribute to loss of meaning in life (Derkx et al. 2020; Sjöberg et al. 2018). For the participants it resulted into questioning and weighing the value of their present lives.

Remarkedly, at the time the interviews were held, all participants explicitly stated that they did not have a wish to die. At the same time, they were also expressing that not waking up in the morning would be an ideal way of dying. The question arises how the expression ‘not waking up in the morning’ should be understood and how participants understood a wish to die. The different ways and tones of voice in which participants talked about this thought points to the possibility that this expression can be seen as a way of saying that the most important part of life has been lived and as a first tentative expression of a latent wish to die. Because zest for life also encompasses a will to live, a loss of zest may influence the will to live. In this way, the scenario of not waking up in the morning may be understood as an expression of a diminishing will to live (Glasberg, Pellfolk, and Fagerström 2014).

In addition, although at two moments during the selection process the participants expressed a WTD, the absence of the WTD at the moment of the interview aligns our study with other research that emphasize the ambivalent nature of a wish to die (Ohnsorge, Gudat, and Rehmann-Sutter 2014; Van Wijngaarden et al. 2021). It can not only change in intensity or stability but it can also diminish or disappear. Moreover, our study adds that not only in context of terminal illness but also in the context of aging it is difficult to understand what expressing a WTD mean, why it is expressed and what function it may have. For example, it may address certain needs, it can be a vehicle to talk about the end of life or it can be a manner to manipulate (Ohnsorge, Gudat, and Rehmann-Sutter 2014).

The absence of a WTD during the interviews contrasts previous research of the authors in which the lived experience of older people who felt that life was completed and no longer worth living was illustrated (Van Wijngaarden, Leget, and Goossensen 2015; Van Wijngaarden, Leget, and Goossensen 2016). Although overlapping themes between these two groups were found especially concerning loneliness and sense of not mattering, in that study participants expressed a WTD. A key difference between the two groups that might (partly) explain this contrast is that in the previous study participants ‘experiences showed disconnectedness from life where as our participants still seemed willing to connect with their lives by questioning its value and looking for meaningful commitments.

## Strenght/limitations

As far as we know, our study is the first study into the lived experience of older persons who see no future for themselves and although they had no WTD, the findings contribute to the knowledge of a possible development of wishes to die in the context of aging. A strength of this study is that by giving voice to experiences of participants who were recruited in the general population, insight is gained into ways older people in the Netherlands live with the future closing down on them.

A limitation is the fact that all participants were Dutch and member of a research panel. Although this panel is an iso certified representative among the general population, the fact that they were a member of this panel may contribute to a selection bias that cannot be excluded. Their willingness to participate in the interview study may also contribute to selection bias. The fact that all participants live within Dutch society where a certain openness regarding aging and end-of-life debates is familiar raises some concerns regarding transferability to societies in which these topics are more tabooed.

The HADS-D was used as a screening tool to explore indications whether participants could be suffering from depression. Because of this explorative aim we did not use the complete HADS. Therefore, its outcome should be interpreted with cautionand not as an accurate indicator of serious mental health issues.

Despite these limitations, this study gives insight into the experiences of older people who see no future for oneself in the context of aging that can provide a starting point for further research. For example, how in ‘seeing no future for oneself’ may manifest itself in different societies and how having or losing a partner may contribute to this experience.

In addition, it would be worthwhile to conduct research among older people who do see a future for themselves and who do experience zest for life with a special interest in how zest for life relates to meaning in life.

## Conclusions

As their horizon of future possibilities is shrinking, older people in our study experience a loss of zest for life and start to questioning the value of their present lives. They face the difficulties of aging by keeping on going and perceive these experiences as something they just live with, not something to take action on.

So, the questioning of present life can be interpreted as a central aspect of seeing no future for oneself in the context of aging. It is essential to further explore what this questioning means, what underlying needs are to be discovered and how these needs should be met.

Our findings illustrate that the phenomenon ‘seeing no future for oneself’ does not entail a wish to die and that caution is required when discussing and making policy around assisted dying for this group.

## Note

1 These numbers do not add up to 34 because some participants mentioned more than 1 health problem.
